# Intensive end-of-life care in acute leukemia from a French national hospital database study (2017–2018)

**DOI:** 10.1186/s12904-022-00937-0

**Published:** 2022-04-02

**Authors:** Sébastien Salas, Vanessa Pauly, Margaux Damge, Veronica Orleans, Guillaume Fond, Régis Costello, Laurent Boyer, Karine Baumstarck

**Affiliations:** 1 Department of Palliative Care, Oncology, APHM, Marseille, France; 2 Department of Medical Information, APHM, Marseille, France; 3 CEReSS - Health Service Research and Quality of Life Center, Aix-Marseille University, EA 3279, 27 bd Jean Moulin, cedex 05, F-13385 Marseille, France; 4 Hematology and Cellular Therapy Department, APHM, Marseille, France; 5 Department of Epidemiology and Health Economics, APHM, Marseille, France

**Keywords:** Acute leukemia, End-of-life care, Palliative care, Registry database, Health services research

## Abstract

**Background:**

A better understanding of how the care of acute leukemia patients is managed in the last days of life would help clinicians and health policy makers improve the quality of end-of-life care. This study aimed: (i) to describe the intensity of end-of-life care among patients with acute leukemia who died in the hospital (2017–2018) and (ii) to identify the factors associated with the intensity of end-of-life care.

**Methods:**

This was a retrospective cohort study of decedents based on data from the French national hospital database. The population included patients with acute leukemia who died during a hospital stay between 2017 and 2018, in a palliative care situation (code palliative care Z515 and-or being in a inpatient palliative care support bed during the 3 months preceding death). Intensity end-of-life care was assessed using two endpoints: High intensive end-of-life (HI-EOL: intensive care unit admission, emergency department admission, acute care hospitalization, intravenous chemotherapy) care and most invasive end-of-life (MI-EOL: orotracheal intubation, mechanical ventilation, artificial feeding, cardiopulmonary resuscitation, gastrostomy, or hemodialysis) care.

**Results:**

A total of 3658 patients were included. In the last 30 days of life, 63 and 13% of the patients received HI-EOL care and MI-EOL care, respectively. Being younger, having comorbidities, being care managed in a specialized hospital, and a lower time in a palliative care structure were the main factors associated with HI-EOL.

**Conclusions:**

A large majority of French young adults and adults with acute leukemia who died at the hospital experienced high intensity end-of-life care. Identification of factors associated with high-intensity end-of-life care, such as the access to palliative care and specialized cancer center care management, may help to improve end-of-life care quality.

**Supplementary Information:**

The online version contains supplementary material available at 10.1186/s12904-022-00937-0.

## Background

Despite the progress made in recent decades, cancer, including hematological malignancies, remains the leading cause of premature death in adults in France (Institut National du Cancer). Acute leukemia, a heterogeneous group of hematological malignancies characterized by the clonal and malignant proliferation of blasts, is responsible for bone marrow failure. Acute myeloid leukemia is generally diagnosed in patients over 60 years of age (median age 70 years), and its incidence increases with age [1]. Acute lymphoid leukemia is the most common cancer in children and accounts for only 20% of leukemia in adults [2]. Due to the nature of the disease and the complications associated with intensive/aggressive treatments, such as allogeneic haematopoietic stem cell transplantation, patients with acute leukemia endure physical and psychological suffering throughout their treatment and their end-of-life care [3–6]. Although new therapies have emerged in recent years that have prolonged survival, many patients experience relapsed disease and die from their malignancy [1, 7, 8].

Since the 2000s, the quality of end-of-life care was improved with the integration of palliative care (https://www.who.int/cancer/palliative/definition/en/), characterized by a multidisciplinary approach in the management of patients with advanced or progressive chronic disease [6, 9]. However, at the end of life, compared with patients with solid tumors, previous studies showed that patients with hematological malignancies are less likely to access palliative care [4, 10–12], are less likely to enroll in a homecare process or rehabilitation center [13, 14], and receive more intensive and aggressive care treatment ([13, 15–17], Johnston, [18] #32, [19, 20]). Several possible explanations for this finding have been suggested: disease-related complications similar to treatment-related complications, sudden and uncertain transitions to a palliative approach to care, strong bonds between staff and patients, hematologic oncologists who are less comfortable discussing death and dying, and unrealistic clinician and/or patient expectations [11, 15, 17].

Among the previous studies, some report data older than 10 years [4, 11–13, 16], from a single center [4, 12, 16], from heterogeneous individuals [19], or focused on old [21] or young populations [18, 20]. Few of them specifically explored patient- and hospital-related factors associated with intensive care treatments at the end of life. In France, a better understanding of how the care of acute leukemia is managed in the last days of life would help clinicians and health policy makers improve the quality care during this special period, while taking into account the wishes of the patients and their families.

This study provides robust and recent information from the French national hospital database about the intensity of care near the end of life for patients with acute leukemia. The objectives of this study were: (i) to describe the intensity of end-of-life care among adult patients (and adolescents) with acute leukemia who died in the hospital from 2017 to 2018; and (ii) to identify the factors associated with the intensity of end-of-life care.

## Methods

### Data source

This was a French population-level retrospective cohort study based on data extracted from the French national hospital database (Programme de Médicalisation des Systèmes d’Information, PMSI). The PMSI is an exhaustive public and private hospital database inspired from the US Medicare system. The PMSI is based on diagnosis-related groups with compulsory information for each hospital stay including socio-demographic characteristics of patient, diagnoses using the International Classification of Diseases, tenth revision (ICD-10), and procedures using the French classification of medical acts (Classification Commune des Actes Médicaux, https://www.ameli.fr/accueil-de-la-ccam/index.php). -The reliability and validity of PMSI data have already been assessed. This data source was previously used in similar studies [20, 22–24].

### Population

The study population included all patients with acute leukemia aged 18 years and older who died during a hospital stay between January 1, 2017, and December 31, 2018. The patients were identified using the algorithm developed by the French National Cancer Institute (INCa, https://aide.groupepsih.com/docs/pmsi-pilot-cancero/principes-generaux/algorithme-inca/, last access 2021, October, 31th), which was specifically designed to identify cancer patients with routinely collected data (codes of diagnosis, codes of medical acts) (Additional file 1). The selection of patients was based on the ICD-10 leukemia-related codes (Additional file 1). The population included only patients in a palliative care situation defined by at least: 1. one code Z515 during the 3 months preceding death (Z515 can be used by the coders if the patient has a palliative care consultant during the hospitalization but also if the healthcare team considers the patient in a palliative phase); and-or 2. being in a inpatient palliative care support bed during the 3 months preceding death (in France, a palliative bed is a bed in a palliative care unit or a bed in another care unit dedicated to a palliative patient). In France, the palliative teams are only assisting with symptom management (not adressing the intensity of care). The patients with a combination of myeloid and lymphoid leukemia were excluded due to: 1) worse prognosis, 2) heterogeneous therapeutic strategies.

### Endpoints

Intensity end-of-life care was assessed using two endpoints: high intensive end-of-life (HI-EOL) care and Most Invasive End-of-Life (MI-EOL) care. HI-EOL care and MI-EOL care definitions were proposed by Earle et al. [13, 25, 26] and jointly endorsed by the National Quality Forum and the American Society of Clinical Oncology.

HI-EOL care is defined by the occurrence of at least one of the following indicators: 1. at least one intensive care unit (ICU) admission in the last 30 days of life; 2. more than one emergency department admission in the last 30 days of life; 3. more than one acute care hospitalization in the last 30 days of life; or 4. at least one intravenous chemotherapy treatment in the last 14 days of life.

MI-EOL care is defined by the occurrence of at least one of the following indicators in the last 30 days of life: 1. orotracheal intubation; 2. mechanical ventilation; 3. artificial feeding (enteral or parenteral); 4. cardiopulmonary resuscitation; 5. gastrostomy; or 6. Hemodialysis.

### Factors associated with end-of-life care intensity

The factors are detailed in the Additional file 1.

### Statistical analysis

The rates of patients receiving HI-EOL care, MI-EOL care, and each constitutive element are provided. To assess the associations between the two endpoints (HI-EOL and MI-EOL, used as separate dependent variables) and sociodemographic, clinical, and hospital data, univariate and multivariate analyses were performed. Variables selected for the multivariate models were: age classes, sex, year of death, social living area, type of leukemia, allogeneic stem cell transplantation, comorbidities, type of hospital, time in a palliative care structure, time between the patients’ home and the hospital, and length of stay. A generalized estimating equation (GEE) was performed to estimate the parameters while taking into account the intrahospital cluster effect (PROC GLIMMIX procedure model, SAS V9.4). The results are presented as adjusted odds ratios (AORs) and their 95% confidence intervals (95% CIs). Statistical significance was defined as *p* < 0.05. Statistical analysis was performed with SAS 9.4 (SAS Institute).

## Results

### Sample

**Fig. 1:**
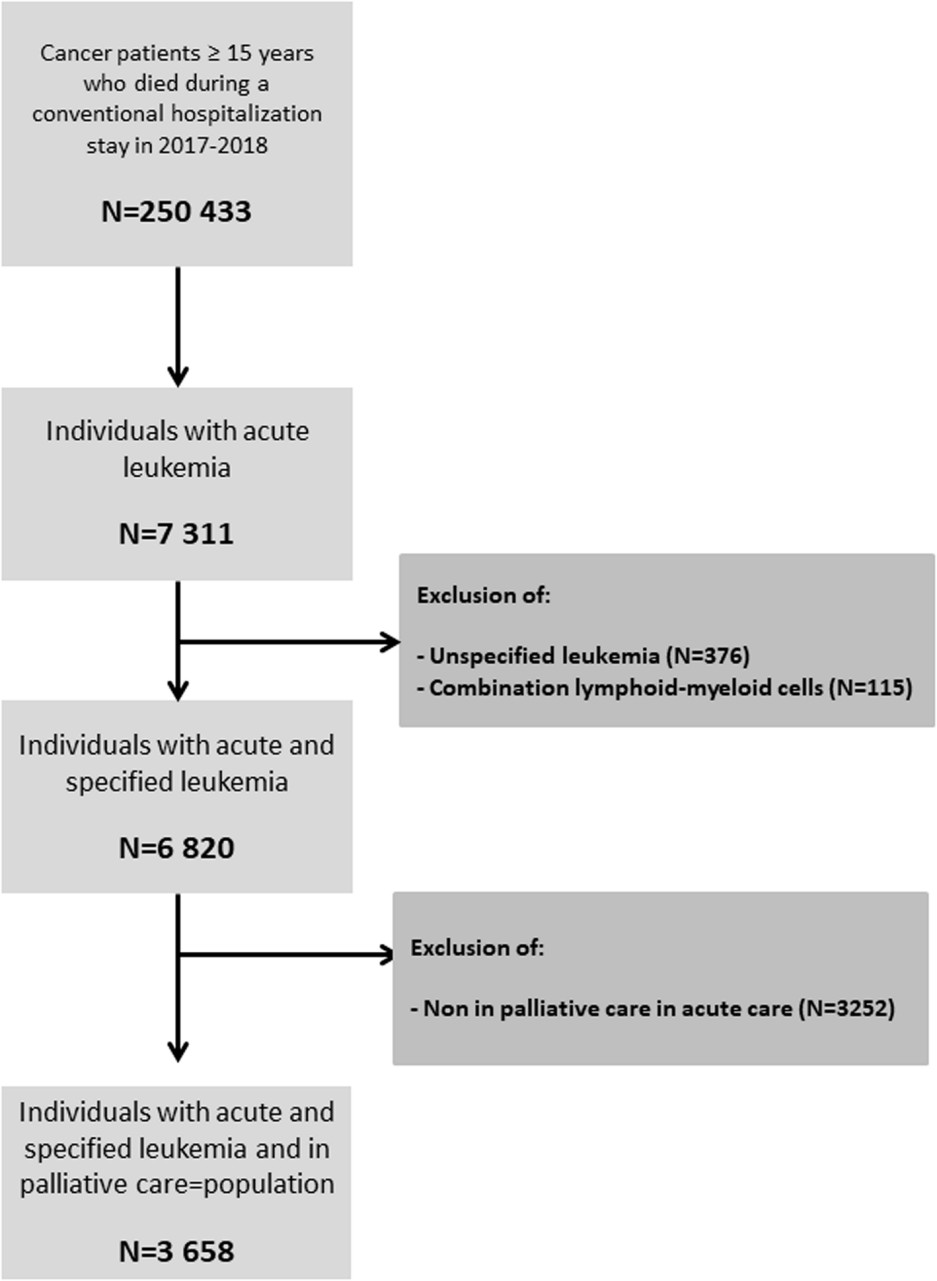
Flow diagram

A total of 3658 patients who died at the hospital between January 1, 2017 and December 31, 2018 were included in the study. We excluded 376 patients with unspecified acute leukemia, 116 patients with a combination of myeloid and lymphoid leukemia, and 3252 patients not in a palliative care situation. A flow diagram detailing the selection of cases is shown in Fig. 1. Twenty-nine percent of the patients were aged under 70 years at the time of death and they were predominantly men (56%). A majority (55%) of the population was living in a socially disadvantaged area. The patients predominantly presented with acute myeloid leukemia (94%) and 7% of them had undergone allogeneic stem cell transplantation. Almost 15% of the patients died in an intensive care structure, 25% of them were hospitalized for more than 1 month, and 30% spent time more than 1 month in a palliative care structure before death. All details are shown in Table 1.

**Table 1:** Socio-demographic, clinical, and hospital characteristics of the 3658 patients

**Socio-demographic data**	**N (%)**
Age at death (years)	
< 60	417 (11.4)
[60–70]	623 (17.0)
[70–80]	1150 (31.4)
≥ 80	1468 (40.1)
Sex	
Male	2037 (55.7)
Female	1621 (44.3)
Year of death	
2017	1813 (49. 6)
2018	1845 (50.4)
Social area living	
Socially advantaged	1608 (44.0)
Socially disadvantaged	2001 (54.7)
Missing data	49 (1.3)
**Clinical Data**	**N (%)**
Type of leukemia	
Acute myeloid leukemia	3426 (93.7)
Acute promyelocytic leukemia	20 (0.6)
Acute myelomonocytic leukemia	170 (4.7)
AML with 11q23	19 (0.5)
AML with dysplasia of several cell lines	161 (4.4)
Acute monoblastic/monocytic leukemia	123 (3.4)
Acute pan myelosis with myelofibrosis	72 (2.0)
Megakaryocyte leukemia	22 (0.6)
Acute erythroid leukemia	31 (0.9)
Acute lymphoid leukemia	231 (6.3)
Months between diagnosis and death	
≤ 3	703 (19.2)
[3–12]	806 (22.0)
[12–36]	1084 (29.6)
> 36	1065 (29.1)
Allogeneic stem cell transplantation	
No	3394 (92.8)
Yes	264 (7.2)
Charlson score (co-morbidities) ^a^	
0	1459 (39.9)
1–2	1411 (38.6)
≥ 3	788 (21.5)
**Last hospitalization stay**	**N (%)**
Type of hospital ^c^	
Non-specialized hospital	2245 (61.4)
Specialized hospital	1413 (38.6)
Death in an intensive care structure	495 (13.5)
Palliative care structure in the last 3 days of life^ b^	745 (20,4)
Time in a palliative care structure before death	
≤ 1 month	2545 (69.6)
> 1 month	1113 (30.4)
Travel time from patients’ home (minutes)	
≤ 10	1047 (28.6)
[10–30]	1071 (29.3)
[30–60]	652 (17.8)
> 60	945 (21.8)
Length of stay (days)	
≤ 15	1382 (37.8)
[15–30]	1361 (37.2)
> 30	929 (25.0)

^a^ Charlson modified score (excluding malignancies/metastasis)

^b^ Intensive care structure includes intensive care unit, ressuscitation unit, emergency unit

^c^ Specialized centers include cancer units of an university hospital and units of a cancer hospital, non-specialized centers include all the other cases

### Intensity of end-of-life care

**Fig. 2:**
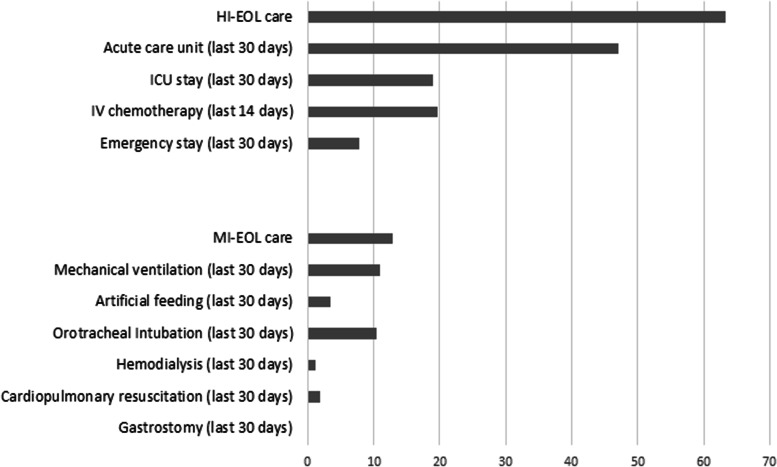
Rates of high intensive end-of-life and most invasive end-of-life care. HI-EOL: high iIntensive end-of-life; MI-EOL: and most invasive end-of-life; ICU: intensive care unit; IV: intravenous

In the last 30 days of life, 63 and 13% of the patients received HI-EOL care and MI-EOL care, respectively. For 20% of the patients, care was managed in an intensive care unit, and 11% of them were mechanically ventilated. All the items constituting HI-EOL/MI-EOL care are presented in Fig. 2.

### Factors associated with HI-EOL care and MI-EOL care

Age, sex, year of death, social living area, type of leukemia, allogeneic stem cell transplantation, comorbidities, type of hospital, time in a palliative care structure, time between the patients’ home and hospital, and length of stay were entered in the multivariate models to assess factors associated with HI-EOL and MI-EOL. HI-EOL care was more often received by younger individuals. The patients with a higher number of comorbidities, hospitalization in specialized cancer centers, a lower time in a palliative care structure, and a longer length of stay were more often associated with HI-EOL care. MI-EOL care was also more often used for younger patients, men, and patients living in a disadvantaged area. The patients with acute myeloid leukemia, a lower time in a palliative care structure, and a longer length of stay were more often associated with MI-EOL care. The multivariate analysis results are detailed in Table 2.

**Table 2:** Factors associated with High Intensive End-of-Life (HI-EOL) and Most Invasive End-of-Life (MI-EOL) care (Multivariate Analyses)

	HI-EOL			MI-EOL		
	***N*** = 2316 (63,31%)			***N*** = 471 (12,88%)		
	N (%)	AOR (Cl 95%)	***p***-value	N (%)	AOR (Cl 95%)	***p***-value
Age at death (years)						
< 60 (ref)	315 (75.5)	–		94 (22.5)	–	
[60;70]	459 (73.7)	0.968 (0.708–1.325)	0.841	124 (19.9)	0.959 (0.686–1.341)	0.807
[70;80]	734 (63.8)	0.688 (0.512–0.926	**0.014**	128 (11.13)	0.558 (0.394–0.791)	**0.001**
> =80	808 (55.0)	0.536 (0.400–0.720)	**< 10**^**−3**^	125 (8.51)	0.445 (0.311–0.635)	**< 10**^**− 3**^
Sex						
Man (ref)	1324 (65.0)	–		278 (13.7)	–	
Woman	992 (62.1)	0.891 (0.770–1.033)	0.125	193 (11.9)	0.940 (0.761–1.161)	0.567
Year of Death						
2017 (ref)	1126 (62.1)	–		220 (12.1)	–	
2018	1190 (64.5)	1.072 (0.928–1.239)	0.346	251 (13.6)	1.104 (0.899–1.356)	0.344
Social area living						
Socially advantaged (ref)	1028 (63.9)	–		204 (12.7)		
Socially disadvantaged	1245 (62.2)	0.944 (0.811–1.100)	0.464	261 (13.0)	1.077 (0.866–1.340)	0.506
Type of leukemia						
Acute myeloid leukemia (ref)	2154 (62.8)	–		426 (12.4)	–	
Acute lymphoid leukemia	162 (69.8)	1.063 (0.773–1.460)	0.708	45 (19.4)	1.216 (0.840–1.760)	0.300
Allo-SCT						
No (ref)	2109 (62.1)	–		400 (11.8)	–	
Yes	207 (78.4)	1.186 (0.832–1.692	0.345	71 (26.9)	1.335 (0.934–1.906)	0.113
Charlson score (co-morbidities) ^a^						
0 (ref)	870 (59.66)	–		149 (10.2)	–	
1–2	939 (66.6)	1.300 (1.104–1.532)	**< 10**^**−3**^	190 (13.5)	1.317 (1.033–1.679)	**0.003**
≥ 3	507 (64.3)	1.142 (0.939–1.89)	0.184	132 (16.8)	1.720 (1.312–2.253)	**< 10**^**−3**^
Type of hospital ^b^						
Non-specialized hospital (ref)	1328 (59.2)	–		217 (9.7)		
Specialized hospital	988 (69.9)	1.357 (1.157–1.592)	**< 10**^**−3**^	254 (18.0)	1.634 (1.312–2.034)	**< 10**^**− 3**^
Time in a palliative care structure						
≤ 1 month (ref)	1611 (63.3)			336 (13.2)		
> 1 month	705 (63.3)	0.577 (0.481–0.690)	**< 10**^**−3**^	135 (12.1)	0.659 (0.509–0.853)	**0.002**
Time from patients’ home (minutes)						
≤ 10 (ref)	617 (58.9)			108 (10.3)		
[10–30]	684 (63.9)	1.224 (1.017–1.474)	**0.032**	131 (12.2)	1.164 (0.79–1.542)	0.289
[30–60]	404 (62.0)	1.114 (0.897–1.382)	0.330	81 (12.4)	1.161 (0.840–1.604)	0.366
> 60	537 (67.5)	1.205 (0.977–1.486)	**0.**081	138 (17.3)	1.335 (0.934–1.906)	0.113
Length of stay (days)						
< 7 (ref)	250 (47.1)	–		46 (10.4)	–	
[7–15]	514 (60.3)	2.114 (1.650–2.709)	**< 10**^**−3**^	81 (10.0)	0.910 (0.608–1.361)	0.645
[15–30]	936 (68.8)	4.053 (3.212–5.114)	**< 10**^**− 3**^	197 (13.2)	1.159 (0.807–1.663)	0.425
> 30	704 (76.9)	7.104 (5.389–9.365)	**< 10**^**−3**^	147 (16.1)	1.474 (0.994–2.186)	0.054

HI-EOL: High Intensive End-of-Life (at least one of the following indicators: 1. at least one intensive care unit (ICU) admission in the last 30 days of life; 2. more than one emergency department admission in the last 30 days of life; 3. more than one acute care hospitalization in the last 30 days of life; or 4. at least one intravenous chemotherapy treatment in the last 14 days of life)

MI-EOL: Most Invasive End-of-Life (at least one of the following indicators in the last 30 days of life: 1. orotracheal intubation; 2. mechanical ventilation; 3. artificial feeding (enteral or parenteral); 4. cardiopulmonary resuscitation; 5. gastrostomy; or 6. hemodialysis)

Allo-SCT: Allogeneic stem cell transplantation

AOR (95% CI): adjusted odd ratio (95% confidence interval)

^a^ Charlson modified score (excluding malignancies/metastasis)

^b^ Specialized centers include cancer units of an university hospital and units of a cancer hospital, non-specialized centers include all the other cases

ref: reference modality for the AOR

Bold values: *p*-value< 0.05

## Discussion

The first important finding of this study was the high proportion of high intensive end-of-life care (63%) during the last days before death in French patients presenting with acute leukemia who died in the hospital. The proportion of most intensive end-of-life care according to the Earle definition was, in contrast, lower (13%). These findings were close to those of studies conducted in similar populations. In the study conducted by Beaussant et al. [19], based on the same French national hospital register but focusing on an older period (2010–2013) and on a larger panel of hematological malignancies (including acute leukemia), some results are relatively consistent with our findings: 26% of patients received a chemotherapy in the last month versus 20% in our results, artificial feeding was used for 7% versus 3% in our cohort, dialysis was used in 5% versus 1% in our study, cardiopulmonary resuscitation was used for less than 2% versus 1.7% in our study. Likewise, in recent studies [18, 20] exploring younger populations (children, adolescents, and young adults with cancer), the authors observed high rates of patients who experienced intensive end-of-life care with significantly higher risk for the group with hematological malignancies. Higher rates were reported in other studies, but they included smaller samples, older patients, and data older than 10 years [16, 21].

These high rates of aggressive end-of-life care may be partially explained. First, in comparison with patients who presented with other malignancies, patients with hematological malignancies (especially patients with acute leukemia) present with a high frequency of hematological complications (bleeding, thromboembolic events, and severe anemia). These complications require emergency treatments, such as transfusions, intravenous antibiotic infusions, and other acute interventions, such that the hospital is often the more comfortable place for treatment administration and monitoring. Second, hematologic oncologists present specificities in their attitudes and beliefs toward end-of-life care. Compared with solid tumor specialists, they are more likely to recommend cancer therapy to patients with poor performance status and short expected survival, and they are less comfortable with end-of-life care [15]. Even if hematologic oncologists report the ability to identify key points in the disease trajectory signifying that end of life is near, they report the absence of a clear definition of the end of life for hematological cancers [15]. Hematologic oncologists report feeling a sense of failure when they are not able to alter the course of disease [15] and have more difficulties sharing prognosis and transition in care with their patients than other oncologists [17]. End-of-life discussions in hematologic oncology often occur too late [17]. It has been shown that focused communication skills training can improve physicians’ abilities to empathically help patients achieve their goals while balancing benefits and burdens [27]. Third, two kinds of aggressive care [17] should be distinguished. Some aspects of intensive care may be considered physician-initiated events (such as hospital admission, chemotherapy administration, intubation) that are influenced by advance care discussions between physicians and patients. Conversely, the decision to present to an emergency unit largely depends on patients and their families themselves and is less likely amenable to physician-driven process intervention. This distinction deserves to be emphasized in future perspectives on improving end-of-life care quality. While some barriers can be reduced with physicians’ interventions, others need discussions with the patients. This highlights the importance of engagement in advance care planning processes [28, 29] that are still insufficient for cancer patients [30].

The second interesting finding relies on the fact that acute lymphoid leukemia patients did not receive more intensive end-of-life care than patients with acute myeloid leukemia, contrary to what other reports found [31]. This result may be partially due to the selection of our sample, that included only patients in a palliative situation.

A third finding drew our attention. A earlier introduction of a palliative care management was a real benefit to protect against aggressive end of life care, as already described in previous studies [32, 33] including randomized trials [9, 34]. Even if having care managed by a palliative structure did not totally protect against invasive/aggressive care during the last days of life [35], earlier palliative intervention had a positive impact on treatment aggressiveness [34]. Nevertheless, as this approach was less often discussed, it is urgent to convince a greater number of centers to plan an earlier transition from curative interventions to palliative care. Qualitative studies may help to identify barriers [36] and provide targeted actions.

Other less expected associations with intensive end-of-life care were found. We hypothesized that the presence of comorbidities, as a reflection of worse health status, would be associated with less intensive end-of-life care, but we found the opposite result. Likewise, compared with nonspecialized cancer centers, we hypothesized that specialized cancer centers, by incorporating multidisciplinary teams, would be less associated with high intensity care [20]. These counterintuitive results may reflect the difficulty of transitioning from curative to palliative objectives. It is now necessary to combine the most advanced technology and therapeutics (such as targeted therapies or allogeneic stem cell transplantation) with consideration of the best end-of-life care quality. Interestingly, the oldest patients were those who benefited least from intensive end-of-life care: this finding was also found in previous reports [20].

### Strengths and limitations

The main strengths are as follows: (1) The study is the first study focusing on acute leukemia in a palliative care situation; and (2) The study includes a large population enrolled in recent years (2017–2018). Some limitations should be discussed: (1) Only patients who died in hospitals were studied, and future studies should explore the phenomenon in other conditions of death, such as patients whose care was managed in rehabilitation centers and at home. This would provide a more valid picture of patients with acute leukemia at the end of life, providing factors associated with the risk of dying in hospitals in France. This may also help improve coordination within the health care delivery system, including specific and general hospitals and ambulatory care. (2) The retrospective design prevented us from truly exploring the relations between the expected prognosis of the patient and the decision regarding intensive care treatments, such as chemotherapy administration, intensive care unit admission, and mechanical ventilation implementation. Physicians have been found to be overoptimistic regarding the prognosis of terminally ill patients [37]. (3) The accuracy of the findings, due to the source of the data (an administrative registry), depends on the coding rules and the skills of the coders [38]. These databases were originally designed for the optimization of funding allocation for the French health facilities, but the coding procedures have moved progressively towards those of a medical record database.

## Conclusion

A majority of French adults, young adults, and adolescents with acute leukemia in a palliative care situation who died at the hospital, experienced high-intensity end-of-life care. Identification of factors associated with intensity end-of-life care, such as the access to palliative care and specialized cancer center care management, may help to improve end-of-life care quality.

## Supplementary Information

**Additional file 1.** Details of materials and methods.

## Data Availability

The datasets generated and/or analysed during the current study are not publicly available: they were used under license for the current study (French national hospital database, the Programme de Médicalisation des Systèmes d’Information) but are available from the corresponding author on reasonable request.

## Abbreviations

AORs: Adjusted odds ratios
EOL: End-of-life
HI-EOL care: High intensive end-of-life care
ICD-10: Classification of Diseases, tenth revision
INCa: French National Cancer Institute
MI-EOL care: Most invasive end-of-life care
PMSI: Programme de Médicalisation des Systèmes d’Information
