# Supplementary material for: Intensive end-of-life care in acute leukemia from a French national hospital database study (2017–2018)
Source: BMC Palliat Care. 2022 Apr 2;21:45. doi: 10.1186/s12904-022-00937-0 (PMC8976296; doi:10.1186/s12904-022-00937-0)
Supplement: Supplementary file 1 — Additional file 1. Details of materials and methods. [file 12904_2022_937_MOESM1_ESM.docx]

**Additional file 1. Details of materials and methods**

**Data source**

The data were extracted from the French national hospital database (Programme de Médicalisation des Systèmes d’Information, PMSI). The PMSI is used to determine financial resources and is thoroughly verified by both its producer and the paying party [Boudemaghe, 2017]. Data from the PMSI database are anonymized and can be reused for research purposes according to French law.

**Selection of patients using the algorithm developed by the French National Cancer Institute**

The algorithm is structured around 8 steps. The 4 first steps are based on the codes of diagnosis. Three definitions of diagnosis are possible: main diagnosis, related diagnosis, and significant associated diagnosis. The 4 next steps allow to refine the targeted patients (with specific codes eliminating invalid situations). The first step is based on the following codes for the main diagnosis : codes malignant tumor (C00-D09 and D37-D48), code chemotherapy for cancer (Z51.1), code chemotherapy (Z51.1) or radiotherapy (Z51.0-) for cancer, codes including the wording ‘related to malignant tumor’ (examples: myasthenic syndrome during a malignant tumor G73.2, cancer treatment K52.0), code bone marrow transplant for malignant tumor (Z94.80-, T86.-). The second step is based on the following codes for the related diagnosis: codes malignant tumor (C00-D09 and D37-D48), codes including the wording ‘related to malignant tumor’, codes bone marrow transplant for malignant tumor (Z94.80-, T86.-). The third step is based on the following codes for the significant associated diagnosis: codes secondary malignant tumor (C77-C79), code of control exam for cancer treatment (Z08), code chemotherapy (Z51.1) or radiotherapy (Z51.0-) for cancer, severe event due antitumoral treatment (Y43.1, Y43.2, Y43.3, Y63.2), code bone marrow transplant for malignant tumor. The fourth step uses a predefined list of medical acts (antitumoral treatment injection, radiotherapy session, brachytherapy session, cell therapy, endoprothesis implementation, organ reconstruction, etc.). The detail is provided here: https://aide.groupepsih.com/docs/pmsi-pilot-cancero/principes-generaux/algorithme-inca/

**ICD-10 leukemia-related codes**

C910, C920, C924-C926, C928, C930, C940, C942, and C944. The ICD-10 codes were validated by two independent expert coders (from the department of medical information). Any discrepancies were resolved by consensus with a third expert coders from the department of medical information.

**Factors associated with end-of-life care intensity**

- Sociodemographic information: age, sex, year of death, and social living area (advantaged vs. disadvantaged area, using the deprivation index; the deprivation index, based on the postal code and validated on French data [Rey, 2009], involves four socioeconomic ecological variables: percentage of high-school graduates, median household income, percentage of blue-collar workers and the unemployment rate).
- Clinical data: type of acute leukemia (myeloid, lymphoid), delay between diagnosis and death, allogeneic stem cell transplantation, chronic comorbid condition based on the modified Charlson comorbidity index [Quan, 2011] (score computed as the number of comorbidities excluding cancer-related items).
- Characteristics of the last hospitalization stay before death: type of hospital (specialized or non-specialized cancer centers; specialized centers include cancer units of an university hospital and units of a cancer hospital, non-specialized centers include all the other cases); death in an Intensive care structure (including intensive care unit, resuscitation unit, emergency unit); transfer to a palliative care structure in the last 3 days of life; time in a palliative care structure before death (excluding home-based palliative care); travel time from the patients’ home to the last hospital; length of stay.

Boudemaghe T, Belhadj I. Data Resource Profile: The French National Uniform Hospital Discharge Data Set Database (PMSI). Int J Epidemiol. 2017;46(2):392-d.

Rey G, Jougla E, Fouillet A, Hemon D. Ecological association between a deprivation index and mortality in France over the period 1997 - 2001: variations with spatial scale, degree of urbanicity, age, gender and cause of death. BMC public health. 2009;9(33).

Quan H, Li B, Couris CM, Fushimi K, Graham P, Hider P, et al. Updating and validating the Charlson comorbidity index and score for risk adjustment in hospital discharge abstracts using data from 6 countries. Am J Epidemiol. 2011;173(6):676-82.
